# Deep sequencing of transcriptomes from the nervous systems of two decapod crustaceans to characterize genes important for neural circuit function and modulation

**DOI:** 10.1186/s12864-016-3215-z

**Published:** 2016-11-04

**Authors:** Adam J. Northcutt, Kawasi M. Lett, Virginia B. Garcia, Clare M. Diester, Brian J. Lane, Eve Marder, David J. Schulz

**Affiliations:** 1 Division of Biological Sciences, University of Missouri-Columbia, Columbia, MO USA; 2 Volen Center and Biology Department, Brandeis University, Waltham, MA USA

**Keywords:** Crustacean, Stomatogastric, Transcriptome, Ion channel, Neurotransmitters

## Abstract

**Background:**

Crustaceans have been studied extensively as model systems for nervous system function from single neuron properties to behavior. However, lack of molecular sequence information and tools have slowed the adoption of these physiological systems as molecular model systems. In this study, we sequenced and performed de novo assembly for the nervous system transcriptomes of two decapod crustaceans: the Jonah crab (*Cancer borealis*) and the American lobster (*Homarus americanus*).

**Results:**

Forty-two thousand, seven hundred sixty-six and sixty thousand, two hundred seventy-three contigs were assembled from *C. borealis* and *H. americanus* respectively, representing 9,489 and 11,061 unique coding sequences. From these transcripts, genes associated with neural function were identified and manually curated to produce a characterization of multiple gene families important for nervous system function. This included genes for 34 distinct ion channel types, 17 biogenic amine and 5 GABA receptors, 28 major transmitter receptor subtypes including glutamate and acetylcholine receptors, and 6 gap junction proteins – the Innexins.

**Conclusion:**

With this resource, crustacean model systems are better poised for incorporation of modern genomic and molecular biology technologies to further enhance the interrogation of fundamentals of nervous system function.

## Background

Despite their status as important economic species, their important place in understanding the evolution and phylogeny of arthropods, and as models for neurobiology research, crustaceans have been largely overlooked in the rush to apply modern molecular biology and high throughput sequencing approaches to work in “non-genetic” systems. Even among arthropods they are fairly poorly represented, with insects dominating the ranks of those with available genome and transcriptome assemblies. Two indicators of this are seen in the number of relative sequence read archive (SRA) and GEO profile publicly available in NCBI: at the time of this article, there were 2,323 crustacean and 46,866 insect SRAs, and 4,608 crustacean and 1,275,029 insect GEO profiles. To date, only two crustacean genomes have been made publicly available [1, 2], with the first, the water flea *Daphnia pulex*, coming only in 2011 [1] – a full 11 years after the first arthropod genome was sequenced [3]. As a microcrustacean, *Daphnia* is still a far cry from the large decapod crustaceans that are common models in neuroscience research, such as crabs, lobsters, crayfish and shrimp. Thus even with some very recent additions to decapod crustacean transcriptome data [4, 5], there is a strong need to add to our sequence knowledge of these species.

Many fundamental findings in neuroscience were made with crustacean preparations. To mention only a small subset of these, command fibers [6], electrical coupling [7] and presynaptic inhibition [8] were first described using crustacean preparations. Work on crayfish and lobsters established GABA as an inhibitory transmitter [9, 10], and allowed early studies of the relevance of the fast outward current, I_A_, for action potential generation and propagation [11, 12]. The first intracellular fluorescent dye-fills were pioneered with crustaceans [13, 14], and crustacean systems were used early on to understand the organization of circuits in behavior [15–18].

Several crustacean circuits, including the stomatogastric nervous system and the cardiac ganglion, continue to provide important new insights into circuit dynamics and modulation [19–22], but this work has been partially hampered by the lack of extensive molecular sequence knowledge in crustaceans. In this study, we generated *de novo* transcriptome assemblies from central nervous system tissue for two commonly used species in neuroscience research: the Jonah crab (*Cancer borealis*) and the American lobster (*Homarus americanus*). We focus on an initial identification, curation and comparison of genes that will have the most profound impact on our understanding of circuit function in these species, namely channels and receptors, with the hopes of fostering new avenues of research for these preparations that continue to be valuable assets in our understanding of nervous system dynamics. Additionally, such sequence information provides a valuable resource for comparative molecular neuroscience approaches across phyla.

## Methods

### Tissue collection and RNA preparation

Adult lobsters, *H. americanus*, and crabs, *C. borealis*, were obtained from The Fresh Lobster Company (Gloucester, Massachusetts, USA) and maintained in chilled (12 °C) artificial seawater tanks until experiments were performed. Lobsters and crabs were anesthetized on ice for at least 30 minutes prior to dissection. The brain, abdominal nerve cord, cardiac ganglion and complete stomatogastric nervous system (STNS) (including the commissural, esophageal, and stomatogastric ganglia) was dissected out of two lobsters and pinned out in a Sylgard (Dow Corning)–coated dish containing chilled (12–13 °C) physiological saline. From two crabs we dissected out brain, complete STNS, and cardiac ganglia. Connective tissue and muscle were removed to the fullest extent possible, and the tissues were rinsed multiple times in chilled physiological saline (Lobster saline composition in mM/l: 479.12 NaCl, 12.74 KCl, 13.67 CaCl_2_, 20.00 MgSO_4_, 3.91 Na_2_SO_4_, 11.45 Trizma base, and 4.82 maleic acid [pH = 7.45]; Crab saline composition in mM/l: 440.0 NaCl, 11.0 KCl, 13.0 CaCl_2_, 20.00 MgCl_2_, 11.2 Trizma base, and 5.1 maleic acid [pH = 7.45]) in ultrapure, RNase-free water. After dissection, tissues for each species were homogenized in Trizol (Invitrogen). The resulting combined pool of RNA therefore consisted of mixed nervous system tissue. Insoluble tissues were pelleted by centrifugation, and the supernatant stored at −80 ° C until RNA extraction. Total RNA was isolated as per the manufacturer’s protocol (Invitrogen), and treated with DNase (Zymo Research) prior to library construction.

### Library construction, sequencing, and *de novo* assembly

Library construction and RNA-sequencing were performed as a fee-for-service by GENEWIZ, Inc. (South Plainfield, New Jersey, USA). Briefly, quantification of RNA samples was performed using a Qubit 2.0 Fluorometer (Life Technologies, Carlsbad, California, USA) and RNA quality checked with an Agilent 2100 Bioanalyzer (Agilent Technologies, Palo Alto, California, USA). Illumina TruSeq RNA library prep, clustering, and sequencing reagents were used throughout the process as specified by the manufacturer (Illumina, San Diego, California, USA). mRNAs were purified using oligo-attached poly-T magnetic beads. The mRNAs were fragmented and first and second strand cDNAs were synthesized and end-repaired. cDNA templates were enriched by PCR following adaptor ligation after adenylation at the 3′ends. cDNA libraries were validated using an Agilent 2100 Bioanalyzer with a High Sensitivity Chip. cDNA library yield was quantified with a Qubit 2.0 Fluorometer (Life Technologies, Carlsbad, California, USA) and by qPCR. After clustering on a flow cell using the cBOT, the samples were loaded on an Illumina HiSeq 2000 instrument for sequencing with 2x100 paired-end reads.

Raw reads were converted into fastq files and de-multiplexed using Illumina CASSAVA 1.8.2. Fastq files were imported into CLC Genomics Workbench Server 5.0.1. Sequence reads were trimmed to remove bases with low quality ends. *De novo* assembly was conducted with the trimmed reads utilizing the CLC Genomics Server. The total length of the assembled transcripts was 66,058,464 bp for crab and 99,847,148 bp for lobster (see Table 1). To ensure that the CLC Genomics assembly was of high quality, we performed a second round of *de novo* assembly using the SeqMan NGen assembler from the DNAstar software suite (SeqMan NGen®. Version 13.0. DNASTAR. Madison, WI.). Following assembly, quality of assembled contigs was investigated by comparison with species-specific sequences contained within GenBank that were previously generated largely by Sanger sequencing approaches. We used BLAST+ command line application (Version 2.2.30+) to perform blastn comparisons of the curated GenBank sequence versus transcriptome contigs, and calculate percent nucleotide identity for the top hit of each sequence for both species.

**Table 1:** Overview of transcriptome assembly statistics for *C. borealis* and *H. americanus*

	*C. borealis*		*H. americanus*	
Raw reads	414,978,768		452,237,240	
Clean reads	391,060,790		426,712,238	
% Q Scores ≥ 30	92.96		92.72	
% GC	43.4		39.4	
Average clean read length (bp)	97.05		97.16	
Assemblers	CLC Genomics	SeqMan NGen	CLC Genomics	SeqMan NGen
Number of Contigs	42,766	67,380	60,273	45,043
N_50_ (bp)	2,178	1,239	2,357	2,258
N_75_ (bp)	1,058	763	1,169	1,085
Mean contig length (bp)	1,544	1,076	1,657	1,799
Longest contig (bp)	21,761	14,125	25,723	17,700
Shortest contig (bp)	454	451	453	450
Total assembled bases	66,058,464	72,508,321	99,847,148	81,065,797

### BUSCO transcriptome quality assessment

To analyze the completeness of our transcriptomes, a reference-based alignment was performed using Benchmarking Universal Single-Copy Orthologs (BUSCO) software (Version 1.22). The arthropod BUSCO reference contains 2675 orthologous genes found within >90 % of the 38 arthropod species’ genomes used to construct the reference [23]. The four transcriptomes we assembled were aligned against the arthropod reference, resulting in percentages of the reference genes found as complete, fragmented, or missing from our transcriptomes. “Complete” genes are those which align to a reference gene with a mean length within two standard deviations (i.e. 95 %) of the reference value. Genes that only partially align are deemed ”fragmented”, and those present in the reference with no match found in the transcriptome are classified as “missing”.

### Functional annotation

For gene ontology (GO) term analysis, the Blast2GO software package (Version 3.1.3) [24] was used for functional annotation of the assembled transcriptomes. A blastx search with an E-value threshold of 10^−5^ was carried out against the NCBI non-redundant (nr) protein sequence database. Assignment of gene names to each contig was based on the highest scoring BLAST hit. Scoring of the annotated sequences utilized a threshold score of ≥ 55. The top 10 significant hits for each query extracted from the blastx search were used for further gene annotation. Query sequences were categorized into three broad ontological classifications: molecular function, cellular component, and biological process. GO annotation filters included: E-value-Hit-Filter of 1.0e-6, Annotation CutOff of 55, and GO Weight of 5.

### Whole-transcriptome alignment comparison

The software VennBLAST [25] was used to compare the whole *C. borealis* and *H. americanus* transcriptomes against the *Daphnia pulex* (GCA_000187875.1) protein sequences from Ensembl Metazoa. Protein sequence database for *D. pulex* was chosen as a common subject to query against the *C. borealis* and *H. americanus* transcriptomes. Initially, a local blastx of *C. borealis* or *H. americanus* contigs against *D. pulex* protein sequences was performed with the BLAST+ command line application (Version 2.2.30+). This output was run through the VennBLAST Merge tool with the InterGroup Option: Use Subject to quantify the relative overlap of *C. borealis* and *H. americanus* with *D. pulex*. A second layer of filtering was performed using the VennBLAST Filter tool with an Identity percent of 70 and an E-value threshold of 1.0e-5, and this output was subsequently merged in the same manner mentioned previously.

### Ion channel and receptor sequence identification and alignment analysis

We identified putative orthologs of channels and receptors from the transcriptomes of crab and lobster as follows. We created local blast databases from the assembled contigs of each transcriptomes. Because channels and receptors are fairly well conserved across diverse taxa, and because the mouse research community has agreed upon a well-curated systematic naming system for channel and receptor genes, we used mouse reference mRNA sequence for each gene of interest as the query in a tblastn search of each transcriptome database. We used initially stringent e-value cutoffs (1e-100 to 1e-50) for our searches to find very high sequence similarity matches. Top contig matches from these blast searches were then compared with the results of the remainder of the blast queries for a given gene family. Often multiple mouse input sequences resulted in the same top hit from the crustacean transcriptomes, indicating that fewer members of the gene family were present in our invertebrate sequence than the mammalian gene families. Once a complete gene family search was obtained, all putative orthologs were then blasted against the Non-Redundant Protein (NR) Sequence Database hosted at NCBI via blastx. This allowed us to look for conserved sequence across all taxa and confirm a given gene identification. Once gene families were obtained from the *C. borealis* transcriptome, the process was repeated with *H. americanus* as the subject database. We additionally used the crab sequences as queries to find the direct ortholog for a given gene in *H. americanus*. These sequences were confirmed in the same way via blastx against the NR database, and moved forward into sequence alignment as described below. As is to be expected from manually performed sequence-by-sequence discovery and curation such as this, at times other searches and sequence comparisons were performed on a case-by-case basis with comparator species such as *Daphnia pulex*, *Drosophila melanogaster*, or other insect species to gain insight or clarification as to the best possible identification for a given transcript.

The web-based software tool Biology Workbench (Version 3.2) [26] was used for sequence analysis of putative ion channels and receptors from the assembled transcriptomes. Coding regions were determined based on the longest open reading frame (ORF) from the SIXFRAME tool in Biology Workbench. ClustalW was utilized (default parameters) to perform the multiple sequence alignment (MSA) for ion channel and receptor family subtypes based on amino acid sequences from predicted coding regions. The rooted phylogenetic trees were constructed from the output of the MSA from ClustalW. The data matrix for all phylogenetic trees was deposited into TreeBASE (Study Accession URL: http://purl.org/phylo/treebase/phylows/study/TB2:S19948).

We used blastp to generate percent identities and similarities for predicted amino acid sequences of orthologs between species. We used only sequences that were full-length or those that were near full length. Sequences were assumed to be full length coding sequences if they met three criteria: the sequence began with a start codon, was approximately the same length as similar sequences in the non-redundant database based on a blastx search, and the sequence ended with a stop codon. Sequences were considered to be close to full-length if they were at least 80 % the length of similar sequences on the NR database, regardless of the presence of start and stop codons. In addition, the two sequences from crab and lobster had to be at least 80 % of the length of one another. This generated 42 pairwise comparisons for orthologous protein sequence between crabs and lobsters.

## Results

We note that all nomenclature for transcripts described in this study will conform to one nomenclature convention: transcripts from *Cancer borealis*, will be noted with the species prefix *Cb-*, and the species prefix *Ha-* will be used for *Homarus americanus* gene products. All curated gene sequences described below were submitted to GenBank and assigned individual accession numbers as noted in Tables 2, 3, 4 and 5.

**Table 2:** Accession numbers for ion channels identified from transcriptome assemblies of *C. borealis* and *H. americanus*

Channel Family	Gene Name	Current/Channel Type	*C. borealis*	*H. americanus*
Voltage-dependent K+ Channels	*shaker*	Voltage-gated A-type potassium (I_A_ or Kv1)	FJ263946	KU702655
	*shab*	Voltage-gated delayed rectifier (I_Kd_ or Kv2)	DQ103255	KU702656
	*shaw1*	Voltage-gated delayed rectifier (I_Kd_ or Kv3)	KU681456	KU681443
	*shaw2*	Voltage-gated delayed rectifier (I_Kd_ or Kv3)	KU681455	KU681444
	*shal*	Voltage-gated A-type potassium (I_A_ or Kv4)	DQ103254	KU702654
	*KCNQ1*	Voltage-gated slow delayed rectifier (M-type or Kv7)	KU681453	KU681441
	*KCNQ2*	Voltage-gated slow delayed rectifier (M-type or Kv7)	KU681452	KU681440
	*KCNH1/EAG*	Ether-a-go-go type potassium (Kv10)	KU681458	KU681446
	*KCNH2*	Ether-a-go-go-related potassium (elk or Kv12)	KU681459	KU681447
	*KCNH3*	Ether-a-go-go-related potassium (erg of Kv11)	KU681460	KU681448
Other K+ channels	*BKKCa*	Large conductance (BK) voltage/Ca^2 +^ −activated potassium	DQ103256	KU712072
	*SKKCa*	Small conductance (SK) Ca^2 +^ −activated potassium	KU710383	KU712071
	*KCNT1*	Sodium-activated potassium	KU681454	KU681442
	*IRK*	Inward-rectifier potassium (IRK)	KU681451	KU681439
	*KCNK1*	Two-pore domain leak potassium (K2p)	KU681438	KU681450
	*KCNK2*	Two-pore domain leak potassium (K2p)	KU681437	KU681449
Ca2+ Channels	*CaV1*	L-type high-voltage-activated (HVA) calcium	N809809	KU702651
	*CaV2*	P/Q-N high-voltage-activated (HVA) calcium	JN809808	KU702650
	*CaV3*	T-type low-voltage-activated (LVA) calcium	JN809810	KU702652
Na + Channels	*NaV*	Voltage-gated fast sodium *para* type (Nav)	EF089568	KU702653
	*NALCN*	non-selective sodium leak	KU681457	KU681445
Hyperpolarization-Activated/Cyclic Nucleotide Gated Channels	*HCN/IH*	Hyperpolarization-activated cyclic nucleotide-gated	DQ103257	KU712077
	*CNG-Alpha1*	Cyclic nucleotide-gated channel alpha 1	KU716097	KU712074
	*CNG-Alpha2*	Cyclic nucleotide-gated channel alpha 2	KU716098	KU712075
	*CNG-Alpha3*	Cyclic nucleotide-gated channel alpha 3	KU716099	KU712076
	*CNG-Beta1*	Cyclic nucleotide-gated channel beta 1	KU716096	KU712073
Transient Receptor Potential (TRP) Channels	*TRP-A1*	Transient receptor potential cation channel, subfamily A, member 1	KX037435	KX037441
	*TRP-A-like*	Transient receptor potential cation channel, subfamily A, member	KX037434	KX037440
	*TRP-M1*	Transient receptor potential cation channel, subfamily M, member 1	KX037436	-
	*TRP-M3*	Transient receptor potential cation channel, subfamily M, member 3	KX037433	KX037439
	*TRP-M-like*	Transient receptor potential cation channel, subfamily M, member	KX037437	KX037444
	*TRP-V5*	Transient receptor potential cation channel, subfamily V, member 5	KX037438	KX037445
	*TRP-V6*	Transient receptor potential cation channel, subfamily V, member 6	-	KX037443
	*TRP-pyrexia*	Pyrexia transient receptor potential channel	-	KX037442

**Table 3:** Accession numbers for biogenic amine and GABA receptor subtypes from transcriptome assemblies of *C. borealis* and *H. americanus*

Receptor Family	Gene Name	*C. borealis*	*H. americanus*
Octopamine/Tyramine Receptors	*Tyr-R*	KU710373	KU712061
	*Oct-αR*	KU710375	KU712062
	*Octβ-R1*	KU710372	KU712063
	*Octβ-R2*	KU710374	KU712064
	*Octβ-R3*	KU710370	KU712065
	*Octβ-R4*	KU710371	KU712066
Dopamine Receptors	*D1αR*	KU710377	KU712059
	*D1βR*	KU710376	KU712060
	*D2αR*	KU710378	KU712058
Serotonin Receptors	*HTR1A*	KU710381	KU712070
	*HTR1B*	KU710382	KU712069
	*HTR2B*	KU710380	KU712067
	*HTR7*	KU710379	KU712068
Histamine Receptors	*HisR1*	KU716100	KU716104
	*HisR2*	KU716101	KU716106
	*HisR3*	KU716102	KU716103
	*HisR4*	-	KU716105
GABA Receptors	*mGABAr-1*	KU986868	KU986874
	*mGABAr-2*	KU986869	KU986875
	*LCCH3-like*	KU986871	KU986878
	*RDL-like*	KU986872	KU986876
	*GRD-like*	KU986873	KU986877

**Table 4:** Accession numbers for glutamate and acetylcholine receptor subtypes from transcriptome assemblies of *C. borealis* and *H. americanus*

Receptor Family	Gene Name	*C. borealis*	*H. americanus*
Metabotropic Glutamate Receptors	*mGluR1*	KU986879	KU986885
	*mGluR2*	KU986880	KU986887
	*mGluR3*	KU986881	KU986888
	*mGluR4*	KU986882	KU986890
	*mGluR5*	KU986883	KU986886
	*mGluR7*	KU986884	KU986889
Kainate-Like Receptors	*Kainate-1A*	KX016772	KX016777
	*Kainate-1B*	KX016773	KX016778
	*Kainate-2A*	KX016774	KX016779
	*Kainate-2B*	KX016775	KX016780
	*Kainate-2C*	KX016776	KX016781
NMDA-like Receptors	*NMDA-1A*	KX016782	KX016787
	*NMDA-1B*	KX016783	KX016788
	*NMDA-2A*	KX016785	KX016789
	*NMDA-2B*	KX016786	KX016791
	*NMDA-2-like*	KX016784	KX016790
Glutamate-Gated Chloride Channel	*Glu-Cl*	KX059698	KX059699
Acetylcholine Receptors	*mAChR-A*	KX021822	KX021833
	*mAChR-B*	KX021821	KX021832
	*nAChR-alpha1*	KX021828	KX021840
	*nAChR-alpha2*	KX021827	KX021839
	*nAChR-alpha3*	KX021829	KX021841
	*nAChR-alpha4*	KX021830	KX021842
	*nAChR-alpha5*	KX021824	KX021836
	*nAChR-alpha7*	KX021825	KX021837
	*nAChR-alpha8*	KX021831	-
	*nAChR-alpha10*	-	KX021835
	*nAChR-alpha16*	KX021826	KX021838
	*nAChR-beta1*	KX021823	KX021834

**Table 5:** Accession numbers for Innexin subtypes from transcriptome assemblies of *C. borealis* and *H. americanus*

	Gene Name	*C. borealis*	*H. americanus*
Innexins	*INX1*	JQ994479	KM984498
	*INX2*	JQ994480	KM984499
	*INX3*	JQ994481	KM984500
	*INX4*	KJ642222	KM984501
	*INX5*	KJ817410	-
	*INX6*	KJ817411	KM984502
	*INX7*	-	KM984503

### Mixed nervous system transcriptome sequencing and de novo assembly

**Fig. 1:**
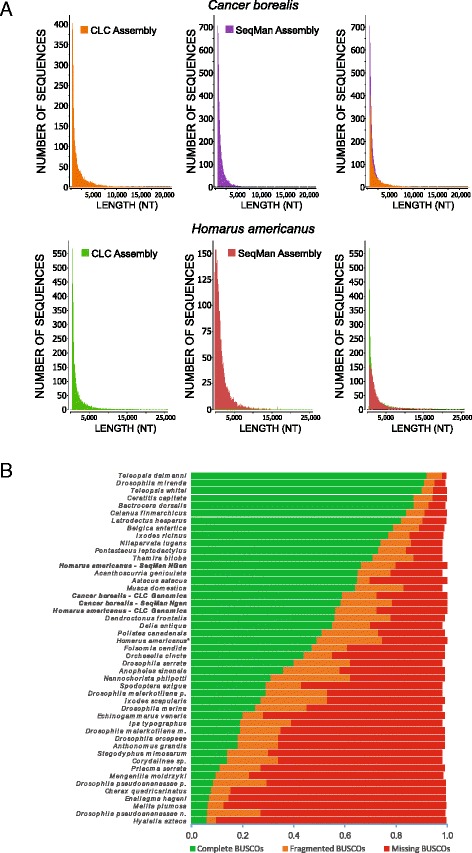
Length distribution of the *de novo* assemblies and annotation coverage of the *C. borealis* and *H. americanus* transcriptomes. **a** Size distribution of contigs shown for two different *de novo* assemblies of the *C. borealis* and *H. americanus* nervous system transcriptomes. Each assembly is shown individually, and overlaid contig lengths are shown in the *right panels*. Assembly statistics are shown in Table 1. **b** *Horizontal stacked bar plots* showing proportions of gene sets in BUSCO quality categories for the 4 different assemblies shown in panel A (CLC and SeqMan NGen, noted in *bold*). A previously published nervous system transcriptome from *H. americanus* (denoted by *) is also provided for comparison [5]. Quality categories are as follows: i) Complete BUSCOs: genes that match a single gene in the BUSCO reference group; ii) Fragmented BUSCOs: genes only partially recovered with gene length exceeding alignment length cut-off; iii) Missing BUSCOs: non-recovered genes

Constructing RNA-seq libraries from nervous tissues of adult crustaceans, a total of 414,978,768 and 452,237,240 raw reads were obtained from the paired - end sequencing of *C. borealis* and *H. americanus,* respectively. The average read length for both species was approximately 97 bp, as expected for 100 bp paired-end Illumina Sequencing. Following quality checks removing adaptors, contaminating sequences, and low-quality sequences, 391,060,790 (94.2 %) clean reads were found for *C. borealis* and 426,712,238 (94.4 %) for *H. americanus*. These high-quality cleaned reads were subsequently assembled *de novo* into contigs using two different assemblers: CLC Genomics and Seqman NGen. For *C. borealis,* CLC assembly resulted in 42,766 contigs with an average length of 1544 bp and an N50 length of 2178 bp, while SeqMan assembly resulted in 67,380 contigs with an average length of 1076 bp and N50 of 1239 (Table 1; Fig. 1a). For *H. americanus,* CLC assembly resulted in 60,273 contigs with an average length of 1657 bp and N50 length of 2357 bp, while SeqMan NGen resulted in 45,043 contigs with an average length of 1799 bp and N50 of 2258 (Table 1; Fig. 1a).

To compare the quality of our transcriptomes from multiple assemblies, a reference-based alignment was performed using BUSCO [23]. The arthropod BUSCO reference contains 2675 orthologous genes expected within most arthropod species that were compared against the gene content of our transcriptomes. The alignment of our transcriptomes against the arthropod reference resulted in similar percentages of the reference genes found as complete (C), fragmented (F), or missing (M) across our transcriptomes (Fig. 1b). The *C. borealis* metrics were C: 59.0 %, F: 13.5 %, and M: 27.5 % for the CLC Genomics assembly and C: 58.4 %, F: 20.1 %, and M: 21.5 % for the SeqMan NGen assembly. The *H. americanus* metrics were C: 56.1 %, F: 16.4 %, and M: 27.5 % for the CLC Genomics assembly and C: 66.5 %, F: 13.4 %, and M: 20.1 % for the SeqMan NGen assembly. These results were compared against the arthropod transcriptome reference scores provided in the BUSCO supplementary materials, a recently published *Homarus americanus* nervous system transcriptome assembled using Trinity [5], and a recent transcriptome of the freshwater crayfish *Astacus astacus* [29]. Our results are comparable with the *Astacus* transcriptome in completeness and an apparent extension of the published *Homarus americanus* transcriptome [5]. One possible explanation for the missing arthropod genes from our transcriptomes can be explained by the fact that our sequences were derived solely from nervous system tissue, while the references were built from arthropod genomic sequences.

Using the NCBI BLAST+ suite to perform a blastn of 28 *Cancer borealis* sequences already contained within GenBank against our assembled contigs, we found an average sequence identity from the CLC assembly of 99.2 %, with the lowest identity score 96 %. SeqMan NGen assembly for *C. borealis* transcripts had an average sequence identity of 99.03 %, and the lowest percent identity was 95 %. For *H. americanus*, 75 GenBank sequences were aligned against our transcriptome, resulting in an average sequence identity to CLC assembled sequences of 99.3 % with the lowest being 94.5 %. SeqMan NGen assembly for *H. americanus* transcripts had an average sequence identity of 98.97 %, and the lowest percent identity was 87 %.

Based on the relative similarity in many of the metrics for these two assembly methods, the somewhat better performance of CLC contigs when compared with Sanger sequencing generated orthologs, and the fact that portions of the *H. americanus* transcriptome based on the CLC assembly have previously been published [4], we chose to perform the remaining representative analysis of these sequence data based on the CLC assembled contigs. The *H. americanus* Transcriptome Shotgun Assembly (TSA) project has been deposited at GenBank under Accession No. GEBG00000000 (BioProject No. PRJNA300643; BioSample No. SAMN04230440). The *C. borealis* Transcriptome Shotgun Assembly (TSA) project has been deposited at GenBank under the Accession No. GEFB00000000 (BioProject No. PRJNA310325; BioSample No. SAMN04450329). The versions described in this paper represent the first versions, GEBG01000000 and GEFB01000000 respectively.

### Annotation and gene ontology mapping

**Fig. 2:**
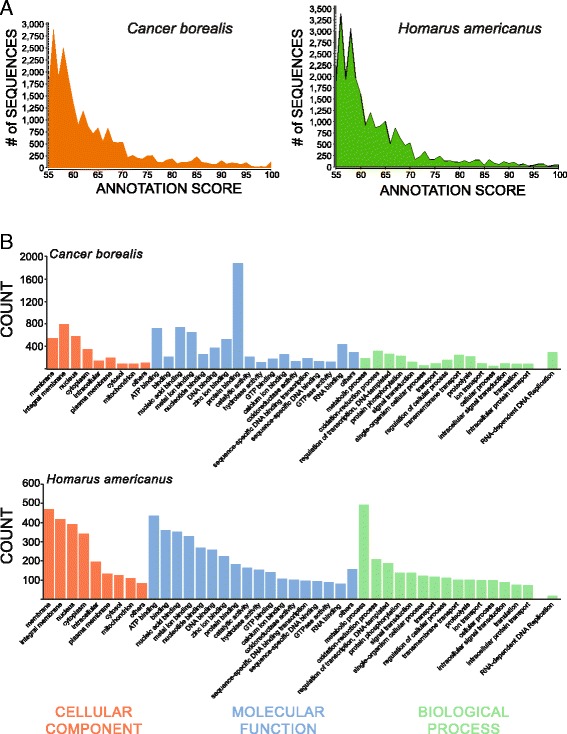
Annotation-score distribution of *C. borealis* and *H. americanus* transcripts*.* **a** Distribution of annotation scores for all Gene Ontology (GO) terms assigned during the Blast2GO annotation process of the CLC assembled contigs. **b** Distribution of GO terms for *C. borealis* and *H. americanus*. Absolute values of GO annotation for the ontology categories of Cellular Component, Molecular Function, and Biological Process. Order was based on top GO counts for *H. americanus,* except for one case (RNA-dependent DNA Replication) due to high incidence in *C. borealis* biological process

**Fig. 3:**
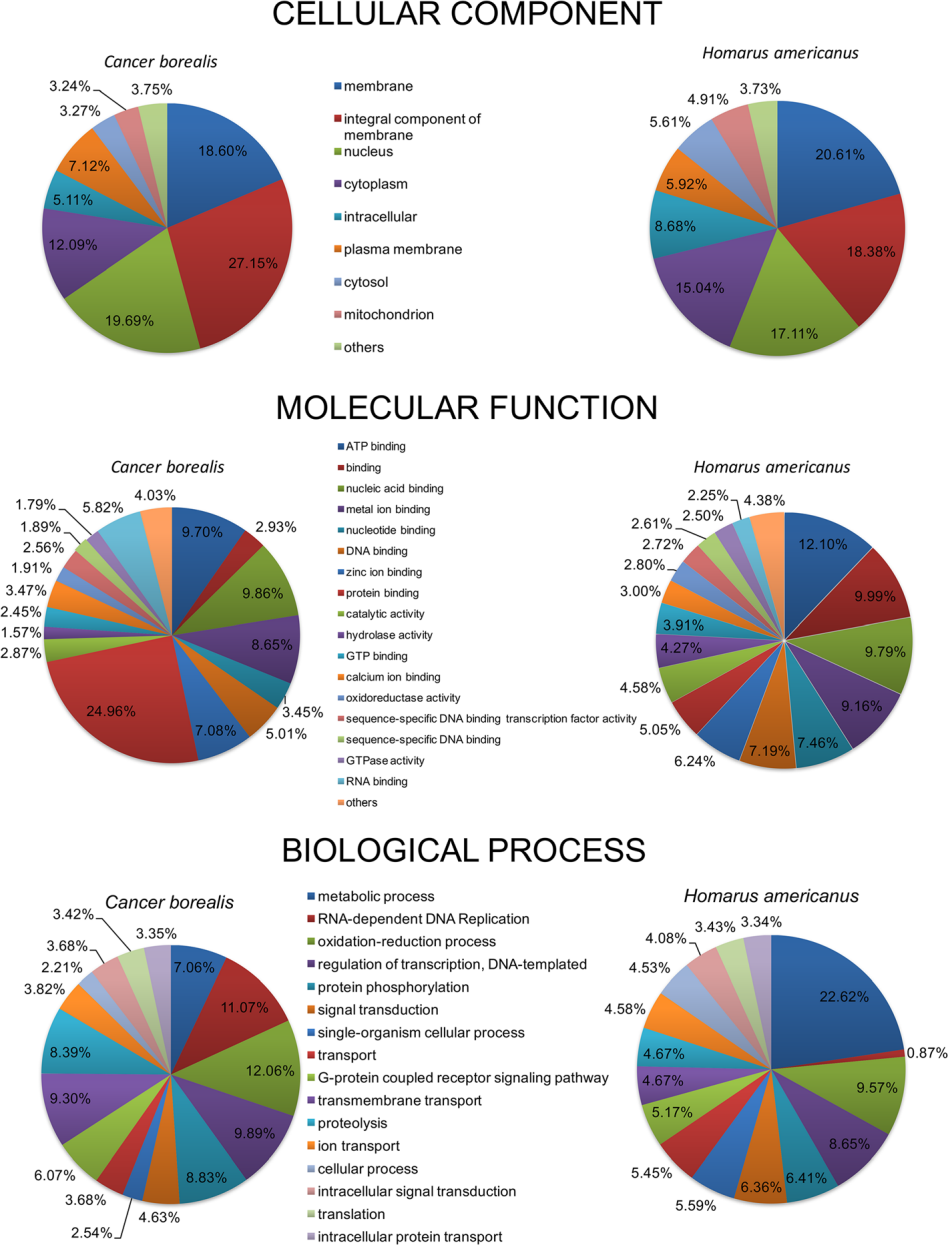
Gene ontology comparison between *C. borealis* and *H. americanus* neural transcriptomes. GO annotation categories Cellular Component, Molecular Function, and Biological Process were plotted as a percentage of their total annotation counts for each category

Entrez Gene IDs were obtained for both transcriptomes using blastx against the NCBI non-redundant (NR) protein database. These annotations consisted of 9489 unique proteins among *C.borealis* transcripts, and 11,061 among *H, americanus* transcripts. Mapping these gene IDs to Gene Ontology (GO) categories yielded 9351 (22 %) of the *C. borealis* and 6191 (10 %) of the *H. americanus* contigs successfully identified (Fig. 2a). Similar percentages have been observed in other *de novo* transcriptome analyses [27, 28]. From the functional annotation, transcripts were classified into three broad categories: cellular compartment (CC), molecular function (MF), and biological process (BP) [24]. Within these broad categorizations, the highest abundance GO terms of *H. americanus* and *C. borealis* were compared against each other, which included the top 9 CCs, 18 MFs, and 16 BPs for both species (Fig. 2b). The arrangement of GO terms was based on the highest abundance *H. americanus* terms, in descending order. The only notable exception to this order was the BP GO term “RNA-dependent DNA Replication” ontology due to its high abundance in *C. borealis* but relatively low abundance in *H. americanus.* These same GO terms were compared between the two species using the relative percentage of each GO term for its broad GO classification (CC, MF, BP) (Fig. 3). The most striking differences between the GO ontologies of each species include a much higher incidence of “protein binding” terms for *C. borealis* MF than that of *H. americanus,* a much higher incidence of “metabolic process” in *H. americanus* BP, and a prominent difference between the “RNA-dependent DNA Replication” term for BP. The source of these differences could be attributed to factors including, but not limited to, the variation in tissue types (abdominal nerve cord was used in *H. americanus,* but not *C. borealis*), depth of sequencing, or natural variation in transcript abundance.

### Species comparisons: distribution and VennBLAST analysis

**Fig. 4:**
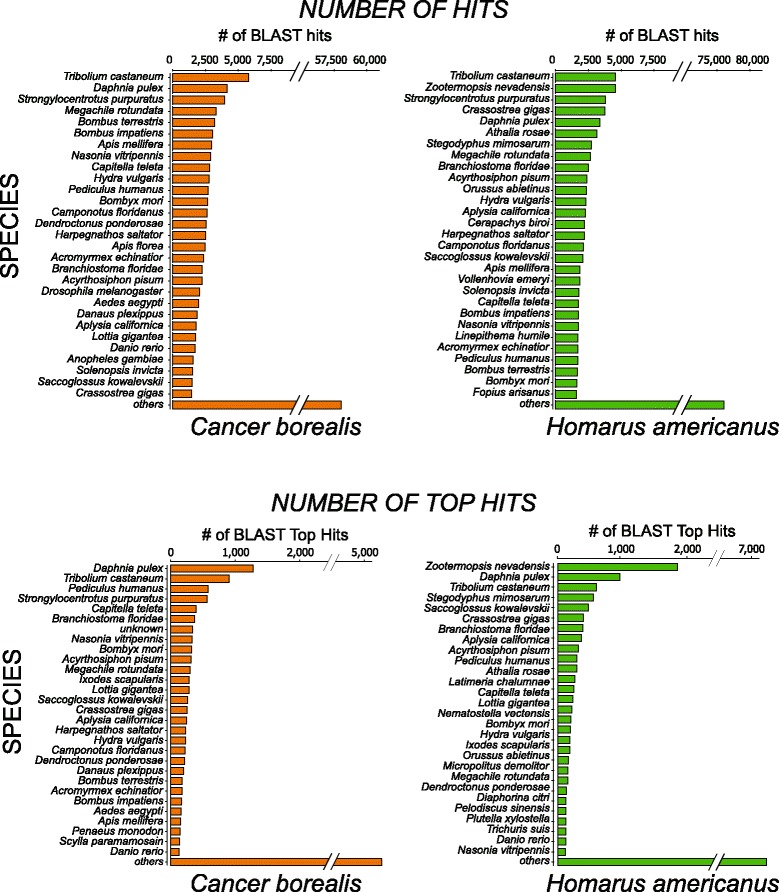
Species distribution of blast hits of *C. borealis* and *H. americanus* neural transcriptomes. Total hits and top-hit numbers for a given species from *C. borealis* and *H. americanus* transcriptomes

Using the Blast2GO software suite, the number of species that the *C. borealis* and *H. americanus* neural transcriptomes align with was determined from a blastx against the NCBI non-redundant database. The species distribution for both *C. borealis* and *H. americanus* gave similar top species, such as *Tribolium castaneum, Daphnia pulex,* and *Strongylocentrotus purpuratus* within the top 5 species hits (Fig. 4). The absence of termite (*Zootermopsis nevadensis*) from the *C. borealis* species distribution of blast hits is due to the fact that the *Z. nevadensis* protein sequences had yet to be uploaded to the NCBI non-redundant database at the time of the blast analysis of the *C. borealis* transcriptome, while the *H. americanus* analysis was performed after the *Z. nevadensis* reference became available.

**Fig. 5:**
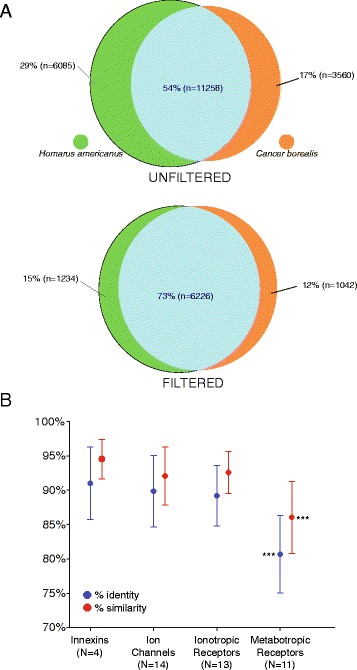
Comparison of overlap of *C. borealis* and *H. americanus* neural transcriptomes. **a** VennBLAST comparison of *C. borealis* and *H. americanus* neural transcriptomes. Alignment of top hit sequence comparison was performed with a tblastx of both *C. borealis* and *H. americanus* against a common top hit species, *D. pulex*, allowing for a highly annotated crustacean database for reference. Filtering added another further stringency on top of that from the tblastx by requiring an amino acid identity percent of 70 % and *E*-value threshold of 1.0e-5. **b** Percent amino acid sequence identity (*blue points*) and similarity (*red points*) for selected neural function related gene products. For the specifically curated gene products described in the remainder of the study, we found very high (>90 %) amino acid sequence identity and similarity between *C. borealis* Innexins (gap junction proteins), ion channels, and ionotropic receptors and the corresponding sequence in *H. americanus*. We saw a significant drop (one-way ANOVA with post-hoc *t-*tests) in similarity in sequences for metabotropic receptor subtypes. This indicates that channel proteins (including gap junction, voltage-gated, and ligand gated) show more highly conserved amino acid sequence than receptors that work via intracellular signal transduction cascades. *** indicates significant difference (*P* < 0.001, *t-*test) between metabotropic receptors and each of the other three groups. None of the other groups were significantly different from one another

Venn diagrams were generated (Fig. 5a) using the software VennBLAST [25] to compare the whole *C. borealis* and *H. americanus* transcriptomes against the *Daphnia pulex* (GCA_000187875.1) protein sequences from Ensembl Metazoa. The protein sequence database for *D. pulex* was chosen as a common subject to query the *C. borealis* and *H. americanus* transcriptomes against due to the mutual high top-hit species distribution (Fig. 4), as well as the well-annotated genome of the crustacean *D. pulex* [1]. Initially, a local blastx of *C. borealis* or *H. americanus* contigs against *D. pulex* protein sequences resulted in 17,343 and 14,818 hits, respectively. Upon overlapping these hits with the VennBLAST Merge tool, 11,258 hits from *C. borealis* and *H. americanus* were found to have the same top hit for *D. pulex*. A second analysis with increased stringency was performed using the VennBLAST Filter tool to retain only high-quality matches, leaving *C. borealis* with 7,460 and *H. americanus* with 7,268 hits to *D. pulex.* Subsequent merger of these filtered hits resulted in 6,226 common top-hits for *D. pulex*, resulting in an increased percentage (from 54 % overlap to 73 %) of common top-hits.

For the remainder of our transcriptome analysis, we identified and characterized sequences for 6 different Innexin proteins (gap junctions), 34 distinct ion channel types, 17 biogenic amine receptors, 5 GABA receptors, and 28 major transmitter receptor subtypes including glutamate and acetylcholine receptors. These are described in detail below. These receptor groups consisted of 27 different ligand-gated channel subunits (ionotropic receptors) and 23 metabotropic receptor types. To better quantify the similarity across lobster and crab, we performed analyses of percent amino acid identity and similarity between orthologs of a subset of genes (see Methods). Overall, the sequence similarity is very high between these species, as one might expect for members of the same Order (Fig. 5b); across 42 genes surveyed, there was a mean ± SD of 85.27 % ± 8.46 % amino acid identity between genes. However, we also noticed a significant trend across different types of gene products: there was significantly lower amino acid identity and similarity for metabotropic receptors than for the other classes of genes (Fig. 5b). In particular, the G-protein coupled (GPCRs) receptors that we analyzed were some of the most divergent between crab and lobster. For example, *Octβ-R1, 3,* and *4* shared 72, 72, and 74 % amino acid identity respectively. Conversely, the most highly conserved genes were in the Shaker family of voltage-gated K^+^ channels. *Shaker, Shal,* and *Shaw1*were 98, 98, and 96 % identical between crabs and lobsters. From these results we would predict more conservation in channel function and physiology across species than that of the GPCRs.

### Ion channels

**Fig. 6:**
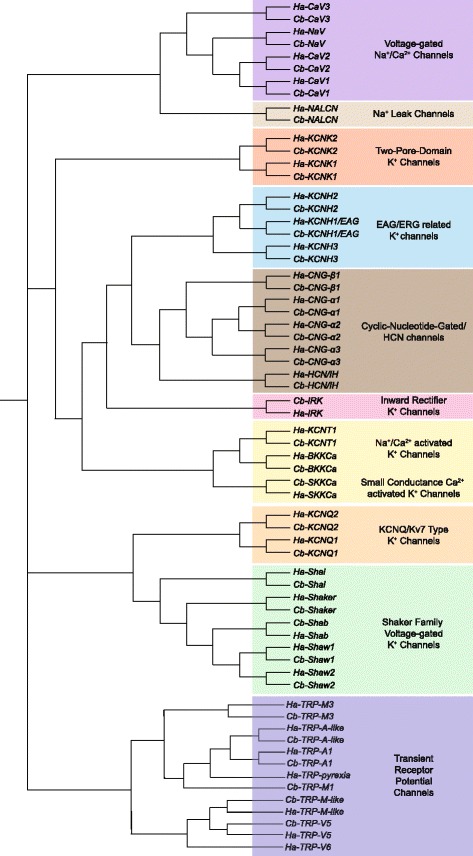
Ion channel subtypes and families identified in *C. borealis* and *H. americanus* transcriptome assemblies. Amino acid sequence alignment was carried out using ClustalW, and subsequent trees were generated using ClustalW2_Phylogeny. We were not attempting to generate true phylogenetic relationships, but rather simply used tree-based analysis to identify putative gene families. Hence no bootstrap values are calculated. There was a 100 % overlap in identified genes from both species, with the exception of TRP channels. A comprehensive list of channel types identified, their putative membrane currents, and accession numbers are provided in Table 2. Transcripts with the prefix “*Cb”* were identified from *C. borealis,* while those with “*Ha*” were identified from *H. americanus*

For our initial analysis of these crustacean transcriptomes, we decided to focus on some of the most critical proteins involved in nervous system function. We therefore first conducted an analysis of ion channel subtypes. Putative ion channels were identified based on tblastx searches utilizing the transcriptomes as a reference database and querying with known channel protein largely consisting of sequences from mouse (*Mus musculus*) and *Drosophila melanogaster*. A 100 % overlapping set of ion channels were found to be present in both *C. borealis* and *H. americanus* (Table 2; Fig. 6). We specifically hand-curated and annotated these channel sequences, and the full list is available in Table 2, including putative current types carried by each channel. We used the multiple sequence alignment (MSA) output from CLUSTALW [30] to develop a fairly comprehensive ion channel tree based on amino acid sequence similarity, allowing us to cluster channels by type to effectively interrogate the nervous system channel content of these crustaceans.

Our analysis of the crab and lobster transcriptomes led us to identify and characterize 34 distinct channel subtypes representing several gene families (Table 2; Fig. 6). Each identified channel transcript was found in both crab and lobster transcriptomes. Our analysis confirmed the presence of 3 major voltage-dependent calcium channel subtypes [31] corresponding one each to the L-type (*CaV1*), P/Q-type (*CaV2*), and T-type (*CaV3*) families of calcium channels. In addition, we identified a single member of the NaV-type voltage-gated Na^+^ channel representing the *para* type channel identified in other species. Finally, in both species we identified a non-selective sodium leak channel (*NALCN*) thought to underlie TTX-resistant Na^+^ conductance important in baseline neuronal excitability [32]. One other major family of non-selective cation channels we identified was the cyclic-nucleotide-gated channels of the HCN/CNG type. Both species contained one member of the hyperpolarization-activated cyclic nucleotide-gated (*HCN*) channel family, the channels that give rise to I_H_ type currents in crustacean neurons [33]. In addition, we identified 3 α -subunits and 1 β-subunit of the cyclic nucleotide gated ion channel types (*CNG*), which are activated by the binding of cAMP and cGMP to carry a non-selective cation current [34].

The pore-forming α-subunits of K^+^ channels can be sub-divided into voltage-dependent subunits (K_v_), inward rectifiers (K_ir_), two-pore subunits (K2P), and those activated by intracellular calcium (K_Ca_) or sodium (K_Na_) ions. We identified a diverse array of voltage-dependent potassium channel subtypes in the transcriptomes of both the crab and lobster (Table 2; Fig. 5). The best already characterized of these channels in crustaceans are the Shaker family of channels, having been identified in crab [35, 36] and spiny lobsters (*Panulirus interruptus*, [37, 38]), with the latter having an extensive characterization via expression in oocyte systems [39, 40]. Previously, 4 members of this family were already known from *Cancer borealis*: *shaker, shal, shab,* and *shaw.* We found orthologs to each of these in *H. americanus* as well. We further discovered that in both species there actually were 2 distinct *shaw*-related channel transcripts (Fig. 6). The newly re-named “*Shaw1*” transcript from this analysis is a perfect match with the previously identified *shaw* transcript from *Cancer borealis* (Accession #EF089569), while the newly identified *shaw*-like transcript is presented as *Shaw2*. We also identified two members of the *KCNQ* family of K^+^ channels. *KCNQ* genes encode a family of six transmembrane domain K^+^ channel alpha-subunits that have a wide range of physiological roles, including likely underlying the slow voltage-gated M-type currents [41]. Rounding out the voltage-dependent K^+^ channel subtypes are 3 members of the *ether-a-go-go*/*KCNH* family. In addition to voltage-dependent K^+^ channels, we also identified one K_ir_ channel (*IRK*), two members of the K2P family (*KCNK*), one sodium-activated K^+^ channel (*KCNT*), and two calcium-activated K^+^ channel types. These K_Ca_ channels had previously been identified in *C. borealis* [36] and correspond to one BK- and one SK- type channel.

Transient receptor potential (TRP) channels have been implicated as a primary channel for generation of sensations including temperature, taste, pain, pressure, and vision. In our analysis, we found various TRP subfamilies within both *C. borealis* and *H. americanus* (Table 2; Fig. 6). These subfamilies included TRPV (vanilloid), mediating odor and pain sensations; TRPA (ankyrin), associated with mechanical stress receptors; TRPM (melastatin), associated with magnesium reabsorption [42]; and TRP pyrexia, a thermal sensing receptor [43]. In crustaceans, TRP channels have been primarily studied for their role in olfactory reception [44] and stretch reception [45]. Not all orthologs of TRP channels were identified in both species. We did not identify in this data set orthologs of *TRP-V6* and *pyrexia* from *C. borealis* and an ortholog of *TRP-M1* was not identified in *H. americanus.* It is most likely that these “missing” orthologs are due to limitations in the sequence depth, although we cannot rule out the possibility that these two species have distinct complements of TRP channel genes. No sequences were found in either species that represent the TRPC, TRPP, TRPL, or TRPN subfamilies. These results are consistent with found in a previously published transcriptome of *H. americanus* that identified 2 TRPA, one *pyrexia,* and two TRPM type channels [5]. We extend these results to include identification of the TRPV family of channels in both *H. americanus* and *C. borealis*.

### Biogenic amine receptors

Biogenic amine neuromodulators were some of the first modulatory compounds to be thoroughly studied in the crustacean nervous system [46–49], and specifically in the stomatogastric nervous system [47, 50–52] where some of the most comprehensive understanding of the multiple targets and modulatory impacts of these compounds on neural circuits has been described [53–59]. Therefore, we decided it would be valuable to provide a thorough characterization of these receptor subtypes as well to complement the extensive and elegant physiology work that has been going on for decades.

**Fig. 7:**
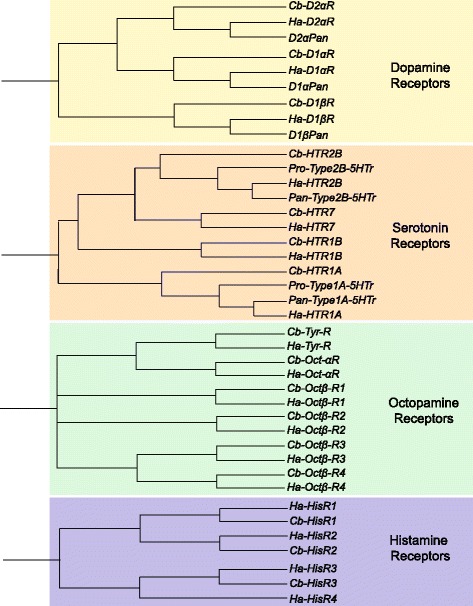
Biogenic amine receptor subtypes identified in *C. borealis* and *H. americanus* transcriptome assemblies. Trees were generated as described in Fig. 6. Once again a 100 % overlap in transcript types was found between the two species, with one exception – a histamine receptor (*Ha-HisR4*) was identified in lobsters that had no corresponding sequence from the crab transcriptome. In the case of serotonin (5HT) and dopamine receptor types, existing crustacean sequence from two different decapod species (*Panulirus interruptus* and *Procambarus clarkii*) were used to help identify orthologs from *C. borealis* and *H. americanus*. These are also included in their respective trees as points of reference. *Pan-* represents *P. interruptus* while *Pro-* represent *P. clarkii.* A comprehensive list of amine receptor subtypes, including accession numbers, is provided in Table 3

Dopamine has been perhaps the most extensively characterized biogenic amine from a functional and biochemical perspective in crustaceans. Previous work [60, 61] identified 3 subtypes of dopamine receptors in the nervous system of the spiny lobster, *Panulirus interruptus*: D1_αPan_ (Type 1A DAr), D1_βPan_ (Type 1B DAr), and D2_αPan_ (Type 2 DAr). We found clear orthologs to all three of these receptor subtypes in both *C. borealis* and *H. americanus* (Table 3; Fig. 7), and our transcriptome search protocol did not come up with any other putative DAr subunit transcripts. Therefore, it is likely that these three receptor subtypes represent the complete complement of dopamine receptors in these decapod crustaceans. In deference to the extensive characterization of these receptors in the closely related spiny lobster, we conform the naming of these channels to match with the *Panulirus* nomenclature: for example, *Cb-D1αR, Cb-D1βR,* and *Cb-D2αR* (Fig. 7).

Serotonin receptors are less described in crustaceans than the dopamine receptors. Previous reports describe two distinct subtypes of serotonin receptors in the *P. interruptus* [62] as well as the crayfish *Procambarus clarkii* [63]: one type-1 and one type-2 serotonin receptor. Our analysis of the transcriptomes of *C. borealis* and *H. americanus* found clear orthologs to both of these receptor subtypes, and based on homology with mouse and *Drosophila* sequences we identified two novel putative serotonin receptor subtypes as well. Figure 6 uses the existing *P. interruptus* and *P. clarkii* sequences to root the new sequences in a tree representing these crustacean serotonin receptors. Our analysis suggests that the previous type-1 5HTr subtypes identified are most similar to mammalian 5HT_1A_ (*HTR1A*) receptors, while the crustacean type-2 5HTr subunit is most similar to 5HT_2B_ (*HTR2B*) receptors. We also identified a putative type-1B receptor (*HTR1B*) as well as a putative type-7 receptor (*HTR7*). These identities are assigned based on mouse query sequences used in our tblastx protocols that generated the strongest hits (i.e. lowest e-values) when queried against the crustacean transcriptomes. We follow the mammalian classification and nomenclature guidelines for these 5HT receptors in assigning gene names (Table 3; Fig. 7), as these are well defined and organized relative to the invertebrate nomenclature: e.g. *Cb-HTR1A, Cb-HTR1B, Cb-HTR2B,* and *Cb-HTR7*.

Octopamine receptors are virtually undescribed in crustaceans, with the sole decapod receptor described as a tyramine/octopamine receptor from the freshwater prawn, *Macrobrachium rosenbergii* [64]. Thorough work with crustacean octopamine receptors is found in the barnacle, *Balanus improvisus*, where one alpha- and four beta-like receptor subtypes have been very nicely characterized [65]. Our analysis identified the same distribution of receptor types in *C. borealis* and *H. americanus* as was described in the barnacle – one alpha- and four beta-like subunits (Table 3; Fig. 7). However, there were no particularly conserved motifs that resulted in a clustering of decapod and barnacle receptor subtypes to converge on a common nomenclature for these receptors; the four beta-like receptors in barnacle most closely related one another rather than subtypes across species. As a result, we have simply named these β-like octopamine receptors subtypes with ascending numbers and in the style of the descriptions given to those identified in *B. improvisus* (*Bi*): e.g. *Cb-Oct-αR, Cb-Octβ-R1, Cb-Octβ-R2, Cb-Octβ-R3,* and *Cb-Octβ-R4*. However, we do not mean to imply direct orthology between *BiOctβ-R1* and *Cb-Octβ-R1*, for example. Finally, we also identified a single putative tyramine receptor in both of our species (*Cb-Tyr-R* and *Ha-Tyr-R*), and these sequences are most similar to the tyramine/octopamine receptor reported from *Macrobrachium rosenbergii*, which is most effectively activated by tyramine [64, 66].

Histamine is also a major neurotransmitter in invertebrates, but sequence information for histamine receptors in crustaceans has yet to be described. In vertebrates, these receptors are part of a protein family of G-protein-coupled metabotropic receptors. In invertebrates, histamine acts exclusively through ionotropic histamine receptors, as no metabotropic histamine receptors appear to be present in invertebrates [67]. We discovered putative orthologs for 3 distinct ionotropic histamine receptors in both *C. borealis* as well as *H. americanus* (*HisR1-3*) and a fourth sequence was found in *H. americanus* that did not have an obvious match from *C. borealis* (Fig. 7). In sequence comparisons with *Drosophila melanogaster*, the closest orthologs are described histamine-gated chloride channels. This is consistent with the known physiological characterization of crustacean histamine gating of chloride channels [68, 69]. Because naming conventions are lacking for these receptors in crustaceans, we have simply assigned these transcripts ascending numbers (1–4) to distinguish between different putative histamine receptor subtypes (Fig. 7; Table 3).

### Metabotropic glutamate receptors

**Fig. 8:**
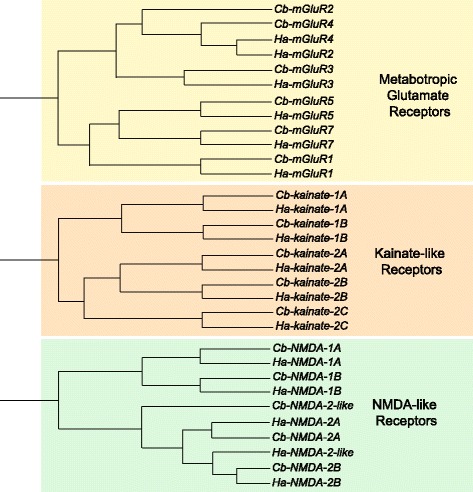
Glutamate receptor subtypes identified in *C. borealis* and *H. americanus* transcriptome assemblies. Trees were generated as described in Fig. 6. We separated glutamate receptor subtypes into metabotropic (G-protein coupled), and the ionotropic Kainate- and NMDA-like. A single glutamate-gated chloride channel (*GluCl*) sequence was identified in both *C. borealis* and *H. americanus*, and is not included as a member of a given receptor subclass in the figure. A comprehensive list of glutamate receptor subtypes, including accession numbers, is provided in Table 4

Metabotropic glutamate receptors (mGluRs) are seven-transmembrane domain proteins coupled to G-proteins capable of controlling many cellular processes through signaling cascades. mGluRs can be classified into three primary classes [70]: Class I consists of mGluR1 and mGluR5 and are associated with phospholipase-C and utilize intracellular calcium signaling cascades; Class II consists of mGluR2 and mGluR3; and Class III consists of mGluR4, mGluR6, mGluR7, and mGluR8 and are negatively coupled with adenylyl cyclase activity. These receptors exist as either homo- or heterodimers on the cell surface, and it is the associated G-protein alpha-subunit that determines which class the mGluR falls under (e.g. Class I associates with G_q_ and G_11_). In crustaceans, mGluRs have a relatively short history of study, mostly owing to the fact that the metabotropic form of glutamate receptors had not been characterized until the late 1980s [71]. In crustacean preparations, it has been found that mGluRs play a role in rhythm generation in the stomatogastric ganglion [72, 73]. In our analysis of these transcriptomes, we found six mGluR sequences for each species, which covered all three primary classes of mGluRs (Table 4; Fig. 8). We did not find mGluR6 and mGluR8 orthologs in either species. It should also be noted that *Ha-mGluR2* and *Ha-mGluR4* aligned more closely to one another than to their *C. borealis* counterparts. This discrepancy could be due to the relatively short partial sequence found for *Cb-mGluR2*; that is, only the first 200 amino acids were found for *Cb-mGluR2*, while the *Ha-mGluR2* sequence found is 1027 amino acids long.

### Ionotropic glutamate receptors

The most common excitatory neurotransmitter found in crustaceans at the neuromuscular synapse is glutamate [74]. The three primary ionotropic glutamate receptors are NMDA, AMPA, and kainate receptors, named respectively after the agonists *N*-methyl-D-aspartate, α-amino-3-hydroxy-5-methyl-4-isoxazolepropionic acid, and kainic acid that activate them. One of the initial characterizations of NMDA receptors from crustaceans was performed in the crayfish optic lobe [75]. Since then, NMDA receptors have been studied in crustaceans for their role in memory [76], axon-to-glial signaling [77], and central pattern generation [78]. We were able to identify 4 separate NMDA receptors from both *C. borealis* and *H. americanus*, which fell into two primary categories: 1-like and 2-like (Table 4; Fig. 8). We further identified these receptors into 1A, 1B, 2A, and 2B based on their pairing, but this is not meant to imply orthology with other naming schemes in other species. We also found one NMDA receptor in both *C. borealis* and *H. americanus* that we did not find a highly similar pair for, which we have included as *Cb-NMDA-2-like* and *Ha-NMDA-2-like*.

AMPA receptors have been virtually undescribed in crustacean preparations, which coincides with our results. In our analysis, we did not find any receptors that most closely resembled AMPA receptors. Known AMPA receptors blasted against our transcriptomes aligned best against the putative kainite-type receptors, which is unsurprising considering both AMPA and kainate are considered non-NMDA receptors. Kainate receptors have been implicated as modulators of synaptic transmission and excitability [79]. In crustacean systems, kainic acid historically has been shown to elicit depolarizations at the crab neuromuscular junction [80], as well as the crayfish neuromuscular junction [81]. Five pairs of kainite-like receptors were found between the *C. borealis* and *H. americanus* transcriptomes (Table 4; Fig. 8), falling into two discrete categories: kainate type-1-like and 2-like. Sequences were further subdivided into A, B, and C on the simple basis of pairing between the two species and not based on any specific orthology to other species.

Beyond the excitatory glutamate-gated cation channels (NMDA- and kainate-like), an inhibitory glutamate-gated chloride channel (GluCl) was also found for both *C. borealis* and *H. americanus* (Table 4). Soon after their discovery as extrajunctional receptors in locust muscles [82], GluCls were described as postsynaptic receptors in the crustacean stomatogastric ganglion [50, 83]. In our analysis, we found a single GluCl transcript for each species, named *Cb-GluCl* and *Ha-GluCl.* The finding of a single channel is consistent with some other invertebrates, with a single GluCl also found in most insects [84]. Because of their distinct characteristics, the GluCl channels we identified were not placed on any of the trees shown in the Figures.

### GABA receptors

**Fig. 9:**
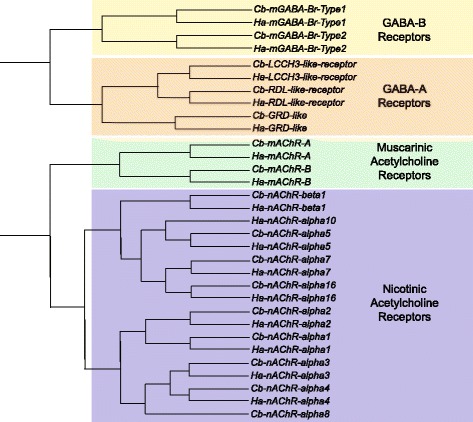
GABA and acetylcholine receptor subtypes identified in *C. borealis* and *H. americanus* transcriptome assemblies. Trees were generated as described in Fig. 6. GABA and acetylcholine are both small molecule transmitters in crabs and lobsters. Both transmitters act through ionotropic and metabotropic receptor subtypes. Metabotropic GABA receptors (GABA_B_-type) and ionotropic GABA subunits (GABA_A_-type) were identified in both species. A comprehensive list of GABA receptor subtypes, including accession numbers, is provided in Table 3. Both nicotinic (ionotropic) and muscarinic (metabotropic) acetylcholine receptors were identified from both species, including one nicotinic beta-subunit and 8 alpha-subunit types. A comprehensive list of acetylcholine receptor subtypes, including accession numbers, is provided in Table 4

The neurotransmitter γ-amino butyric acid (GABA) has been studied in crustacean species for decades for its role in synaptic transmission and neural inhibition [85–88]. Interestingly, in invertebrates several different neural responses to GABA have been found that have distinct profiles from that of vertebrate GABA receptors [83, 89–91]. GABA receptors are classified into two major groups: GABA_A_ type receptors, comprising receptor complexes that are part of a ligand-gated ion channels, or GABA_B_ type receptors, G-protein-coupled receptors that act via metabotropic signaling systems. GABA_A_ receptors are pentameric transmembrane receptors responsible for fast, usually inhibitory synaptic currents, and heteromultimers of the individual subunit types can form distinct channel properties in invertebrates [92]. We identified orthologs of three GABA_A_ type receptor subunits from both *H. americanus* and *C. borealis* (Fig. 9; Table 3), including orthologs of *Drosophila* LCCH3-, RDL-, and GRD-like receptor subunits – and we have preserved naming conventions for these subtypes. Two GABA receptor subunits previously have been cloned from *H. americanus* [93], and very recently a GABA_A_ type receptor was identified in the crayfish, *Procambarus clarkii* [94]. Sequence comparison reveals these previously described sequences to be orthologous to the RDL-like receptor from our data. The metabotropic GABA_B_ type receptors are GPCRs responsible largely for slower inhibitory synaptic effects, and functional GABA_B_ receptors are heterodimers formed by GABA_B1_ and GABA_B2_ subunits [95]. We identified orthologs of both GABA_B1_ and GABA_B2_ subunits in both *C. borealis* and *H. americanus* (Table 3; Fig. 9).

### Acetylcholine receptors

Acetylcholine receptors are classified into two family subtypes: nicotinic receptors (nAChRs), which are ligand-gated ion channels that are activated by nicotine; and muscarinic receptors (mAChRs), which are metabotropic GPCRs that respond to the agonist muscarine. Muscarinic acetylcholine receptors are further classified into subtypes based on the specific G-protein associated with the receptor [96]. In our analysis, we discovered two discrete subtypes of mAChRs (Table 4; Fig. 9) in both *C. borealis* and *H. americanus,* which is consistent with other arthropods [97]. The A- and B-type mAChR are defined based on their differential sensitivity to muscarine (A-type is 1000x more sensitive than B-type), as well as the antagonist binding properties (atropine, scopolamine, and QNB block A-type but not B-type) that each receptor exhibits [97].

Nicotinic acetylcholine receptors are common throughout the invertebrate central nervous system, mediating largely fast excitatory neurotransmission [98–101]. For nAChRs in nervous systems of *C. borealis* and *H. americanus*, each species was found to have 1 β-subtype and 8 α-subtypes (Fig. 9). A thorough characterization of invertebrate nAChRs has been performed for the snail, *Lymnaea stagnalis* [102]*.* Our analysis revealed that few direct orthologous sequences occurred from snail (Mollusca) to crustaceans that would allow us to adopt the nomenclature put forward in *Lymnaea.* Therefore, the crustacean receptor subtypes were named based on their most similar mammalian counterpart, with the exception of the α16 subunit found, named so due to its similarity to *acr-16* found in *C. elegans* [103]*.* This subunit is most comparable to α7 in humans, but we found other sequences nAChRs more similar to human α7 than that of the putative α16.

### Gap junction proteins (Innexins)

**Fig. 10:**
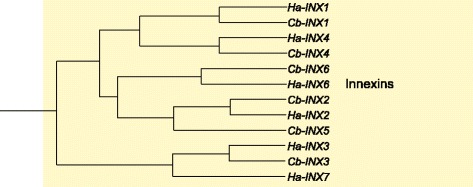
Innexin subtypes identified in *C. borealis* and *H. americanus* transcriptome assemblies. Trees were generated as described in Fig. 6. Innexins are proteins responsible for gap junctions in invertebrates. Six distinct Innexin subtypes were identified in both *C. borealis* and *H. americanus* (see also [103]). Of these six, one from each species did not contain enough sequence homology to classify as the same type across species (*Cb-INX5* and *Ha-INX7*) hence are named as distinct subtypes. A comprehensive list of Innexin subtypes, including accession numbers, is provided in Table 5

The proteins responsible for gap junctions in invertebrates are the Innexins [104]. A family of Innexins have previously been described for both *C. borealis* and *H. americanus* [105]. We include them here as characterized through the transcriptome analysis for completeness. Three full-length sequences were named Innexins 1–3 (Fig. 10; Table 5) based on significant sequence similarity to coding sequences of Innexins from multiple other organisms, including other decapod crustaceans [106]. Innexins 4–6 (Fig. 10; Table 5) were subsequently identified from our transcriptome analysis of *C. borealis* nervous system. We identified clear orthologs from lobster and crab for Innexins 1–4 and 6; but two Innexins showed enough dissimilarity to be classified separately, and these are classified as Innexin 5 in *C. borealis* and Innexin 7 in *H. americanus* (Fig. 10). All of these identified Innexin sequences have the signature motif YYQWV in the second TM domain as well as a series of other conserved amino acid residues considered hallmarks of Innexins [105].

## Discussion

The era of modern genomics and high-throughput sequencing has revolutionized the study of neuroscience, and provided an opportunity for classic physiology systems in the study of neural circuit properties to experience a renewed level of impact. In particular, invertebrate model systems that historically have been invaluable to our understanding of basic circuit properties, dynamics, systems neuroscience, and neuromodulation now present themselves as novel contributors to molecular neuroscience. In particular, classic preparations such as the *Tritonia* swim system [107], *Aplysia* feeding circuits as well as the classic gill and siphon withdrawal reflex [108, 109], and crustacean stomatogastric systems [19] revolutionized our understanding of neural circuitry. Each of these systems is the renewed focus of genomic and transcriptomic approaches [4, 5, 110, 111] – including this study – that promise to merge the unparalleled experimental accessibility on the neurophysiology end of the spectrum with new molecular tools to understand and manipulate these circuits. Decapod crustacean systems have also been foundational in the understanding of modulation of behavioral states. The earliest work implicating serotonin broadly in aggression can be traced to seminal work in lobster behavioral studies [48, 112]. GABA was first identified as an inhibitory transmitter in these same decapod lobster species [9, 113]. Finally, the crayfish escape behavioral response has been a paradigm for the true integration of neuroethological work across single neurons, neural circuitry, behavior, modulation, and social status [114]. Therefore, the accessibility of a molecular perspective and toolset will allow researchers to revisit these seminal works with greater potential to understand integrated nervous system function.

It can be challenging to stay up to date with all of the sequence data being published. To the best of our knowledge, there are a relatively small number of published transcriptome projects with decapod crustaceans as model systems. Sequence discovery projects in decapod crustaceans began with expressed sequence tag (EST) analyses over a decade ago [115, 116], and these have been used to examine olfactory receptor expression in the lobster system [117]. Since then, a mixed tissue transcriptome sequencing projects have been performed from the spider crab, *Hyas araneus* [118, 119] and spiny lobster *Sagmariasus verreauxi* [120]. Some of the most proactive work in crustacean transcriptomics lies in the area of neuropeptidomics for the discovery of neuroactive peptides and their receptors [121–124], including a study that makes use of the *H. americanus* sequence data contained reported here [4]. However, none of these studies provides analysis of ion channel or receptor sequence as contained within our study.

The most directly comparable transcriptome study was carried out in *H. americanus*, and used RNA-seq to examine differences in gene expression across tissues, including nervous system, as well as responses to hormonal treatment and temperature perturbations [5]. While this study uses GO and other annotation approaches to identify channel and receptor groups that are differentially expressed, identification of these gene products is at the level of gene family. For example, McGrath et al. [5] identify glutamate receptors as a broad group that are differentially expressed in tissues, but make no further distinction as to receptor subtype –reporting that they have 126 transcripts that map to a global glutamate receptor subtype group. This high number is likely due to identification of multiple short contigs of the same receptor sequence, as opposed to distinct receptor sequences, and may be reflected by the lower N_50_ value of this transcriptome (1,289). In contrast, the N_50_ of our *H. americanus* transcriptome is 2,357, leading to longer contigs and often the identification of full-length coding sequence that greatly aided our ability to characterize and identify distinct channel and receptor subtypes. Nevertheless, to the extent these studies can be compared, we saw results largely consistent with the overall identifications provided [5]. For example, both studies identified 5 octopamine receptor subunits, including at least 3 beta-type receptors. Further, all reported differentially expressed voltage-dependent ion channel and transmitter receptor subtypes [5] were present in our data set. While there is no direct overlap in the goals of these studies, the characterization provided in our study will provide a reference by which subsequent expression analyses can be combined with thorough annotation to provide more insight into gene-specific changes related to neural function.

## Conclusion

In this study we sequenced the nervous system transcriptomes for two highly utilized species in invertebrate neuroscience research: the Jonah crab (*Cancer borealis*) and the American lobster (*Homarus americanus*). Our sequencing, assembly, and annotation efforts have yielded an extensive set of sequence information from which we can begin to mine gene products critical to fundamental nervous system output: channels and receptors. This study represents the first attempt to characterize to this extent these critical building blocks of circuit function from these model systems. In doing so we have identified for the first time in these species previously undescribed channel and receptor families, as well as added to the incomplete characterization of amine receptors known to modulate both circuit function and behavior in these animals. This sequence information opens up these target proteins for use in gene manipulation techniques such as overexpression [125] or RNA-interference mediate knockdown [126] to deeply interrogate circuit function. Finally, the stomatogastric system has been used extensively in computational studies that have revolutionized our understanding of circuit fundamentals, dynamics, and the role of variability in neuronal parameters in circuit function [127–130]. These models have relied on biological data for identification of likely membrane conductances present in the networks. Molecular screening and quantitative assays of channel expression can effectively be used in concert with computational modeling [131, 132] to generate better and more biologically realistic models with which to uncover fundamental aspects of neural circuit dynamics.

## Abbreviations

5HT: Serotonin
BUSCO: Benchmarking universal single-copy orthologs
BP: Biological process
CC: Cellular compartment
DAr: Dopamine receptor
EST: Expressed sequence tag
GABA: Gamma-aminobutyric acid
GluCl: Glutamate-gated chloride channel
GO: Gene ontology
GPCR: G-protein coupled receptor
mAChR: Muscarinic acetylcholine receptor
mGluR: Metabotropic glutamate receptor
MSA: Multiple sequence alignment
nAChR: Nicotinic acetylcholine receptor
NR: Non-redundant
ORF: Open reading frame
SRA: Sequence read archive
STNS: Stomatogastric nervous system
TRP: Transient receptor potential
TSA: Transcriptome shotgun assembly
